# Evaluation and Treatment of Thoracic Insufficiency Syndrome and Early-Onset Scoliosis

**DOI:** 10.3390/jcm14030753

**Published:** 2025-01-24

**Authors:** Margaret Bowen, Vineet Desai, Jason B. Anari, Patrick J. Cahill

**Affiliations:** 1 Division of Orthopaedics, Children’s Hospital of Philadelphia, Philadelphia, PA 19104, USA; 2 Harvard Medical School, Boston, MA 02115, USA; 3 Perelman School of Medicine, University of Pennsylvania, Philadelphia, PA 19104, USA

**Keywords:** thoracic insufficiency, early-onset scoliosis, dysplasia

## Abstract

Thoracic insufficiency syndrome (TIS) and early-onset scoliosis (EOS) are complex pediatric conditions involving deformities of the spine and chest wall, which can significantly impact respiratory function and overall development. Managing these conditions requires a comprehensive approach that combines precise diagnosis and innovative treatment strategies. This opinion article provides a critical discussion of the diagnosis and treatment of TIS and EOS and reflects upon the advancement of methods that are crucial for assessing these conditions and guiding treatment decisions.

## 1. Introduction

The management of pediatric spine and chest wall deformities has evolved considerably in the 21st century. Thoracic insufficiency syndrome (TIS), defined as the inability of the thorax to support normal respiration or lung growth, can arise from several different underlying etiologies [1]. Three key diagnostic categories that lead to TIS include neuromuscular dysfunction leading to chest and spine deformities, thoracic dysplasia resulting in chest volume and rib cage dysfunction, and spinal deformity causing a distortion and subsequent functional loss of the rib cage [1]. Many patients experience a combination of these conditions contributing to their diagnosis of TIS. Early-onset scoliosis (EOS) is defined as curvature of the spine greater than 10 degrees diagnosed at onset in a child under 10 years old [1]. Managing EOS and TI is challenging and without intervention can lead to significant deformity, worsened cardiopulmonary function and development, and often early mortality [2,3,4,5].

In recent years, there have been significant advancements in technologies, devices, and drug therapies to address rib and spinal deformities. Growth-friendly implants and pharmaceutical agents have revolutionized the management of TIS and EOS, offering new avenues for improving patient outcomes [6]. These advancements not only help in controlling spinal curvature and stabilizing the thoracic cavity but also contribute to better overall respiratory function and quality of life for affected children. As our understanding of the underlying causes of TIS and EOS continues to expand, these emerging therapies offer hope for reducing the long-term impact of these challenging conditions.

References for this manuscript were selected to provide a comprehensive yet focused examination of the key advancements in diagnosing and managing TIS and EOS. The literature was identified through targeted searches of databases such as PubMed and Google Scholar using keywords including “thoracic insufficiency syndrome”, “early-onset scoliosis”, “growth-friendly implants”, and “dynamic imaging”. Priority was given to high-impact studies, recent innovations, and foundational works shaping current understanding. We also sought an array of references that presented diverse perspectives on treatment approaches, highlighting their respective advantages and limitations to ensure a balanced discussion. Inclusion criteria encompassed peer-reviewed publications, seminal studies, and articles that offered critical insights or novel contributions to the field, ensuring the discussion was grounded in authoritative and up-to-date evidence.

## 2. Diagnosis of EOS and TIS

EOS is diagnosed based on the patient’s history, radiographic evaluation, pulmonary status, and clinical exam. In an effort to standardize the wide variety of underlying etiologies leading to EOS, Williams et al. developed and validated the C-EOS. This classification system categorizes EOS based on etiology, major curve angle, and presence of kyphosis [7]. Categories include congenital, neuromuscular, syndromic, and idiopathic scoliosis.

Congenital or structural scoliosis arises from abnormalities or asymmetries in the spine or thoracic cavity, such as hemivertebrae, fused ribs, and a wide range of disorders with underlying abnormalities in bone and/or cartilage development such as Jeune syndrome, VACTERL, Jarcho–Levin syndrome, spondylothoracic dysostosis, and more. Neuromuscular scoliosis typically involves dysfunction in the neurological or muscular control of the trunk muscles and includes patients with either hypotonic or hypertonic neuromuscular conditions, such as static encephalopathy, muscular dystrophies, spinal muscular atrophy, cerebral palsy, spina bifida, and spinal cord injuries. Syndromic scoliosis is associated with specific syndromes such as Marfan syndrome, Ehlers–Danlos syndrome, or down syndrome that may predispose individuals to scoliosis. Finally, idiopathic scoliosis has no identifiable cause, even in cases where there are significant comorbidities that lack a known connection to scoliosis.

Regardless of the etiology of EOS, TIS represents a severe manifestation with significant clinical implications, particularly in terms of its impact on the patient’s health-related quality of life (HRQOL). TIS can be a life-threatening condition in which the worsening deformity of the thorax results in a decline in respiratory function [3,4,8]. There is no gold-standard definitive test or laboratory marker to make the diagnosis of TIS. Thus, the diagnosis is primarily based on clinical evaluation. During a physical exam, the provider may evaluate thoracic size using physical measurements and assess thoracic dynamics with the thumb excursion test, which measures rib cage movement and its ability to expand the thorax. Radiographic parameters are continuing to evolve, with recent studies utilizing dynamic imaging to evaluate lung function [9].

When assessing TIS and deciding whether to pursue operative treatment, key factors to consider include the severity and progression of rib deformity, the patient’s age and skeletal maturity, clinical and objective assessments of pulmonary status, and the degree and rate of progression of spinal deformity [10,11]. There are several diagnostic methods that directly inform the decision to move forward with operative treatment, including pulmonary function testing (PFT), radiographic parameters, and dynamic magnetic resonance imaging (MRI) assessments [12]. PFT is a crucial tool for measuring forced vital capacity (FVC), forced expiratory volume (FEV1), and total lung capacity (TLC) [13]. Radiographic parameters can quantify rib and spinal deformity to further evaluate pulmonary function. All of these tools are valuable for monitoring the progression of deformity and assessing pulmonary decline. Recent studies have found that radiographic variables indicating chest cavity asymmetry are significantly negatively correlated with pulmonary function [14,15,16]. One unique tool used to assess TIS is quantitative dynamic magnetic resonance imaging (QdMRI), which provides a 4D characterization of lung volume changes, illustrating thoracic–abdominal organ structures and their movement. Further development is necessary to establish normative data and improve its integration into surgical planning for more precise patient-specific interventions.

Due to the wide variety and complex medical history of many patients with TIS, a multidisciplinary approach to care and decision-making is vital. Coordination, collaboration, and communication between specialists who treat patients with EOS and TIS allows for a well-rounded perspective and streamlined recommendations in planning treatment. These conversations regarding management of EOS and TIS typically span specialists from neurosurgery, pulmonology, plastic surgery, general surgery, gastroenterology, radiology, and occupational therapy. Each of these perspectives bring important insight into appropriate perioperative care for patients and ensure that patients receive holistic management of both their clinical condition and its effects on their quality of life.

## 3. Advancements in Dynamic Imaging

Clinical metrics have been unable to reliably predict significant improvements in lung function after surgical intervention for TIS [17]. The ability to quantify diaphragmatic motion under free-breathing conditions emerges as a unique avenue of evaluating pulmonary status pre- and post-treatment [12,13]. The recent development of QdMRI, which captures new valuable biochemical and structural parameters, offers more detailed insight into the impact of surgery on lung volume and function. This technique offers the benefit of free-breathing imaging, combined with the creation of an optimized 4D respiratory cycle, which allows for detailed analysis of compartmental changes in lung volume from movements of the chest wall and diaphragm on both sides [9]. These changes can subsequently be evaluated in relation to the function of spine and chest wall pre- and post-treatment [17].

Tong et al. (2019) investigated the application of QdMRI to TIS in pediatric patients. It was found in their investigation that all components of tidal lung volume increased substantially after the implantation of VEPTR constructs [17]. Their data also suggest that thoracic and lumbar Cobb angle are poor predictors of MRI tidal volumes, while pulmonary function measures including FVC and assisted ventilation rating demonstrated moderate correlation with tidal volumes [13,17].

Since the initial application of QdMRI to TIS, there have been several advancements in the technique, particularly with the incorporation of deep learning models. High accuracy has been reported in the interactive segmentation of lung tissue for TIS patients, enabling more precise evaluation of lung function [12,13,14]. Additionally, auto-segmentation techniques for hemi-diaphragms and the thoracoabdominal organs have further refined surgical planning and outcome assessment in pediatric TIS cases, overcoming imaging limitations such as low resolution and motion blur [16,18,19]. These advanced MRI techniques will continue to develop and offer substantial benefits for the care and treatment of the TIS community.

## 4. The Role of Basic Science

Basic science plays a crucial role in advancing our understanding and treatment of EOS and TIS, offering insights into both the biological mechanisms and potential therapeutic strategies. Animal models such as porcine and rabbit models have been shown to evaluate growth-friendly surgical techniques, instruments, and pulmonary function outcomes in EOS and TIS. A rabbit model of early-onset thoracic deformity was investigated by Olson et al. to assess its impact on the growth, structure, and function of the spine, thorax, and lungs [20]. They found that severe thoracic deformity during early development led to reduced lung growth and function, with early treatment potentially preventing pulmonary complications in adulthood. This study highlights the potential of animal models of early-onset thoracic scoliosis as strong pathways to evaluate the growth-friendly surgical techniques and instruments in future growing spine research.

The emerging role of genetics in thoracic insufficiency and EOS is shedding new light on the complex interplay between genetic factors and the broad phenotypic spectrum of these conditions. Despite advancements in next-generation sequencing, the diagnostic success rate varies widely, from 30% to 75%, depending on the specific condition, with lower success rates in isolated scoliosis and higher rates in cases of skeletal dysplasia [21]. Genetic evaluation, alongside comprehensive assessments in pulmonology, ophthalmology, cardiology, nephrology, endocrinology, and developmental health, should be an integral part of managing EOS and TIS, potentially leading to earlier diagnosis and more personalized treatment approaches.

## 5. Surgical Management

In EOS and TIS, when non-operative management fails, operative treatment is generally pursued to prevent deformity progression, enhance sitting posture for non-ambulatory patients, improve patient health-related quality of life, and promote lung development. Historically, patients with progressive EOS have been indicated for early spinal fusion. However, advancements in growth-friendly treatment of EOS now allow for continued spinal growth and thoracic development, such as traditional growing rods (TGRs), Vertical Expandable Prosthetic Titanium Ribs or VEPTRs (DePuy Synthes Spine, Westchester, PA, USA), growth guidance constructs, and, most recently, the magnetically controlled growing rod (MCGR) [6,22]. Growth-friendly surgery is designed to promote maximum thoracic and pulmonary growth until the patient nears skeletal maturity, at which point a definitive spinal fusion can be performed. Research indicates that the use of growth-friendly implants as the initial surgical treatment for EOS has significantly risen over the past two decades [6,23,24]. The choice of surgical approach is determined by the type of EOS or TIS, chest wall anatomy, underlying condition, and any comorbidities. Each respective method offers specific benefits and carries particular risks associated with the chosen approach and device.

Traditional growing rods (TGRs) have been widely used to effectively treat and correct spinal deformity. The indications and technique for this instrumentation have evolved significantly since it was first described by Harrington in 1962 [25,26]. The most common current technique popularized by Akbarnia involves a dual-rod construct with the proximal and distal aspects of each rod connected via a tandem connector [27,28,29]. Anchor site selection is dependent upon the patient’s diagnosis, age, and the nature of the curvature. TGRs require interval distraction, typically every 6 months, in order to promote spinal growth and maintain correction of the deformity. Recent studies highlight higher efficacy of TGRs as distraction techniques for patients with stiff hyperkyphotic curves, congenital scoliosis, and those with short stature [30]. Despite modifications over time, TGRs are associated with a high complication rate, including implant failure, surgical site infections, and wound healing issues. The need for repeated surgical lengthening increases these risks, with reports of up to 58% of patients experiencing complications [22,28,31]. There remains uncertainty when considering TGRs as the implant choice in comparison to other growth-friendly options such as VEPTRs or MCGRs.

Developed by Dr. Robert M. Campbell, the VEPTR revolutionized the management of EOS and TIS. The VEPTR is a titanium alloy device attached at the concave side of the ribs and is distracted as skeletal growth occurs in order to expand the thoracic cage [32,33]. With the primary goal of improving lung volume and function by controlling chest wall deformity, the VEPTR device is lengthened every 6 months and can be attached in several variations, including rib to rib, rib to spine, and rib to pelvis. Depending on the nature of the spinal or chest wall deformity and the underlying diagnosis, surgeons may utilize pelvic anchors for non-ambulatory patients or those with significant pelvic obliquity. While surgical management with VEPTRs has been a common and reliable treatment option over the years, many investigations have revealed high complication rates including implant failure, wound infections, and device migration [34,35,36,37,38,39]. Over the last decade, VEPTRs have been more commonly utilized for congenital or thoracogenic deformity, and studies have suggested that early intervention may benefit TIS patients needing respiratory support, particularly during critical lung growth periods [40]. Some studies suggest that VEPTRs may not provide improvement in TIS but instead delay or prevent deterioration of pulmonary function [40,41,42]. Overall, the use of the VEPTR has declined with the rise of the MCGR.

The MCGR device is composed of an implantable rod which is anchored to the spine with standard fixation components such as pedicle screws and/or hooks. MCGR constructs may be secured proximally at the spine or ribs or distally at the spine or pelvis [43,44]. Patients with MCGR instrumentation undergo periodic distraction using an external remote control, approximately every 3 to 4 months [45]. This treatment option has become increasingly popular as it allows more frequent lengthenings and limits the associated risks and costs of surgical interventions that TGRs and VEPTRs require. By 2014, MCGRs became the most frequently used devices in initial surgeries, surpassing VEPTRs and TGRs [6]. However, further investigations have found the MCGR to have a similar profile to the classic techniques and may pose additional complications, including implant prominence, failure to lengthen, rod breakage, actuator failure, and metallosis [46,47,48].

Many efforts by surgeons, hospital systems, industry, and study groups have attempted to improve upon the complication rate and quality of life of these children with complex deformity. Quality improvement projects along with consensus found in best-practice guidelines have provided hope for families and providers alike aiming for minimal complications during EOS treatment [22,49,50,51,52,53,54,55]. TGRs, VEPTRs, and MCGRs represent an improvement from early fusion and are the prevailing surgical treatment for most types of EOS. However, due to the repetitive nature of distraction-based techniques, complication rates for operative treatment of EOS and TIS remain common [56]. Younger patients, those with syndromes or neuromuscular conditions, and those with higher curve magnitudes or thin skin coverage are at higher risk for complication [57].

## 6. Management of Unique Diagnoses

**Figure 1:**
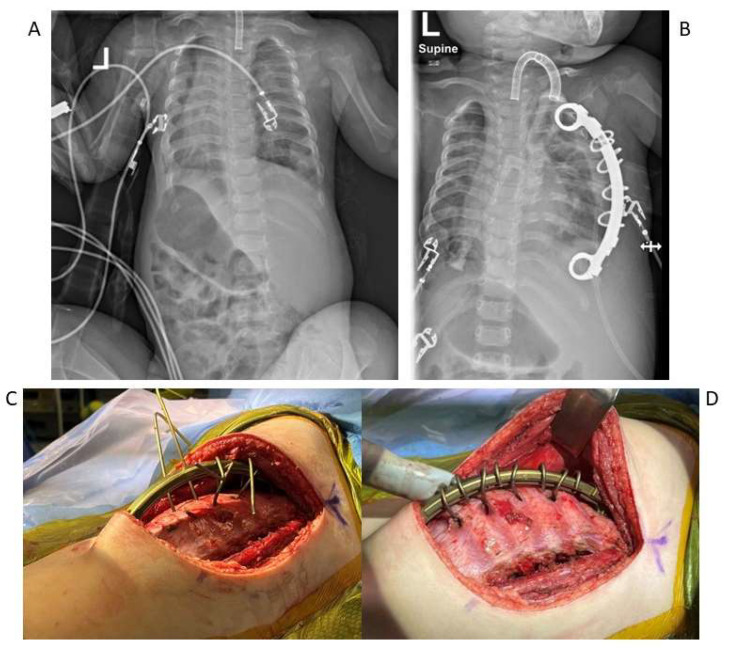
(**A**,**B**) Spine and chest radiographs of a patient with Jeune syndrome showing a preoperative constricted chest cavity and postoperative appearance post staged expanding thoracoplasties with insertion of bilateral rib-to-rib VEPTRs. (**C**,**D**) Intraoperative photos of expanding thoracoplasties with rib-to-rib VEPTR insertion.

Understanding the nuances of spine and chest wall manifestations for each particular underlying etiology of EOS is pivotal for implementing personalized and effective management strategies in EOS and TIS. For example, Jeune syndrome, also known as Asphyxiating Thoracic Dystrophy, is a rare autosomal recessive skeletal dysplasia with multi-organ involvement. Its genetic dysfunction often involves mutations in genes associated with normal skeletal development, resulting in the characteristic skeletal anomalies observed [58]. The most significant characteristic of Jeune syndrome is a narrow chest due to shortened ribs. This can result in a small and constricted thoracic cavity, frequently described as a “three-leaf-clover chest”, leading to TIS and cardiopulmonary compromise (Figure 1). Frequent infections are caused by a combination of small thoracic cages, hypoplastic lungs, and ciliary dysmotility. Severe cases may require respiratory support and the syndrome is often (60–80%) said to be fatal in early childhood [59,60].

Management with VEPTR constructs has played a crucial role in managing Jeune syndrome, particularly when thoracic insufficiency is a primary concern. The main goal of this procedure is to increase thoracic space, promoting lung development and reducing respiratory limitations. In 2015, Campbell reported that the survival rate for Jeune syndrome patients who underwent VEPTR implantation with thoracoplasty was nearly 70%, compared to a 70–80% mortality rate without intervention [60]. Thorough preoperative evaluations and careful postoperative monitoring, including pulmonary function tests and imaging studies, are essential for assessing the surgery’s impact on respiratory health and skeletal development [61].

**Figure 2:**
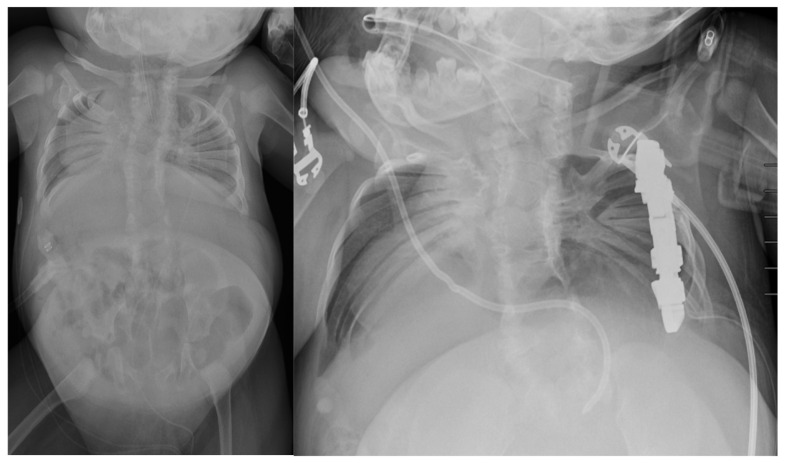
Pre- and post-right rib wedge osteotomy and VEPTR insertion radiographs demonstrating the bilateral fusion of the costovertebral junction in a patient with STD.

Spondylothoracic dysostosis (STD) is a rare congenital disorder characterized by significant spine and rib deformities that impact both skeletal development and respiratory function. Genetic studies have shown that mutations in the MESP2 gene are commonly associated with STD [62,63]. Patients with STD have complete fusion of the costovertebral junctions bilaterally, creating a fan-like or “crab-like” configuration that can be appreciated radiographically (Figure 2) [63]. Mild thoracolumbar scoliosis is sometimes present, along with reduced vertebral numbers and complete cervical spine fusion. This condition limits the use of the chest wall and neck muscles for breathing, leaving patients dependent on their diaphragm. As a result, STD is associated with a high infant mortality and severe respiratory complications, with only a small percentage of patients surviving into adulthood [64].

Surgical management in STD has been a point of controversy throughout the literature and across institutions. Some studies have suggested that thoracoplasty may not benefit patients with STD, while others, including Campbell, demonstrated positive outcomes in these patients treated with bilateral VEPTR central wedge opening thoracostomies [63,64,65]. The perioperative management of patients with STD includes structural and functional assessments such as entire spine MRI, CT scans, cervical spine radiographs in flexion and extension, and pulmonary function testing. Further studies assessing operative versus conservative management are warranted in order to better evaluate decision-making, treatment outcomes, and improve health-related quality of life measures for this population.

Spinal Muscular Atrophy (SMA), a rare neuromuscular disorder that frequently presents with EOS and TIS, presents as another distinct challenge. SMA is an autosomal recessive genetic disorder characterized by a group of inherited conditions that result in the degeneration of anterior horn cells in the spinal cord. This leads to the destruction of alpha motor neurons, causing weakness and atrophy in the proximal muscles [66,67,68].

As defined by Campbell, a unique presentation of chest wall and spine deformity in children with SMA is the “collapsing parasol rib deformity”—in which the rib cage collapses inwards due to weakened intercostal muscles [69]. There is a consequent shrinkage of the chest wall and decrease in room available for lung growth due to collapse of the thoracic cage. In order to predict the likelihood of a patient developing a parasol rib deformity and associated poor pulmonary function, the Parasol Score was developed in 2015. A Parasol Score equal to or below 0.56 defines a parasol rib deformity [69].

$$Parasol\;Score=\frac{T6\;convex\;hemithoracic\;width}{T6\;concave\;hemithoracic\;width}\times \frac{T6\;thoracic\;width}{T12\;thoracic\;width}$$

**Figure 3:**
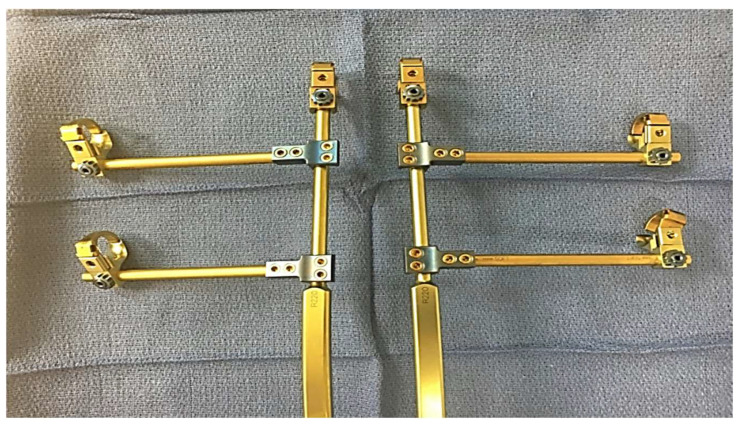
Photo of a VEPTR gantry construct.

**Figure 4:**
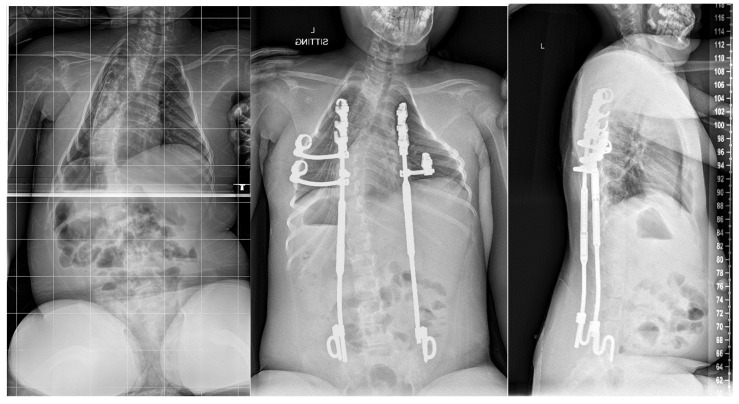
Pre- and postoperative radiographs of an 8-year-old male with Type Ib SMA showing insertion of modified MCGR construct with VEPTR anchors and rib gantries.

While complications remain high for this population, studies have shown the ability of growth-friendly instrumentation to control spinal deformity and maintain pulmonary function [70,71,72,73,74,75]. An ideal treatment of SMA scoliosis not only controls the scoliotic deformity but also controls the parasol rib deformity, avoids repeat surgery, and allows access to the spinal canal. In order to meet these needs and address the downward arching rib pathology unique to SMA, a modified VEPTR construct with rib gantries may be used (Figure 3 and Figure 4).

Historically, SMA had no curative or disease-modifying treatments, and the mortality rate for SMA1 was nearly 100% before the age of two without respiratory support. However, with the advent of new treatments such as nusinersen, risdiplam, and onasemnogene abeparvovec, survivorship rates are improving compared to the previously grim natural course of the disease [76]. The introduction of these three SMN-enhancing treatments for SMA has highlighted the need to review treatment usage among clinicians and patients while further research and evidence are gathered. Children with SMA are now living longer, leading pediatric orthopedic surgeons to manage and treat conditions in a patient population they have not traditionally encountered. This evolving situation requires adaptable treatment options and techniques.

## 7. Conclusions

The management of EOS and TIS has significantly progressed over recent years, with advancements in surgical techniques, growth-friendly implants, and a deeper understanding of the genetic and biological factors underlying these conditions. The introduction of growth-friendly devices has transformed treatment approaches, allowing for better control of spinal and chest wall deformities while promoting lung development and function. Despite these innovations, challenges remain, particularly concerning the high complication rates associated with these treatments, emphasizing the need for ongoing research and refinement in surgical techniques.

The role of a multidisciplinary approach in the assessment and treatment of TIS and EOS cannot be overstated. Collaboration among specialists from various fields, such as neurosurgery, pulmonology, and occupational therapy, is crucial for developing comprehensive and personalized care plans for affected children. As our understanding of the genetic components of TIS and EOS continues to grow, there is hope for even earlier diagnosis and more targeted therapies, potentially reducing the long-term impact of these conditions. Furthermore, the use of animal models and the exploration of new pharmacological treatments provide promising avenues for future research and innovation in this field.

Ultimately, there is a continued need for careful evaluation and adaptation of treatment strategies. The complexity of these conditions demands a holistic and individualized approach to care, with an ongoing focus on minimizing complications and enhancing the quality of life for patients. This era continues to be exciting as providers and scientists work to tackle emerging challenges and improve patient outcomes through innovative techniques and treatments. As the field evolves, the integration of new technologies, genetic insights, and collaborative care models will be key to advancing the management of TIS and EOS in the years to come.
